# Supplementary material for: Predicting students’ satisfaction with academic services at a multicultural engineering university in Bangladesh: A multiple regression analysis
Source: PLoS One. 2024 Sep 6;19(9):e0309223. doi: 10.1371/journal.pone.0309223 (PMC11379188; doi:10.1371/journal.pone.0309223)
Supplement: S1 File — (DOCX) [file pone.0309223.s001.docx]

## **SURVEY TOOL ON STUDENTS’ PREFERENCE OF IUT AS THEIR ACADEMIC CAREER CHOICE**

**Letter to the Student/Respondent**

Dear Student/Respondent,

You have been selected to participate in this study by filling out this Survey with all honesty and sincerity. I am conducting research titled ***“Assessing Predictors of First Year Students’ Levels of Satisfaction with Islamic University of Technology Services: A Multiple Regression Analysis*** The purpose of this study is to scientifically predict factors that influenced first-year students to choose IUT as the University of their career choice. The findings of the study are expected to contribute towards improvement in terms of, teaching & learning processes, students’ support services, students’ campus life, and students’ economic factors.

Please be assured that your responses shall be kept confidential and shall be used only for the purpose of this research.

For further details, please feel free to contact me on my mobile phone +881844 056 016 or on email address [mwebesaumar@gmail.com](mailto:mwebesaumar@gmail.com)

Dr. Mwebesa Umar

**Researcher**

**SECTION A: BIO-DATA**

***(Please tick one that is most applicable to you)***

1. **Sex?**

Male

Female

**2- Age?**

18 – 22 years

23-27 years

28-32 years

33 years and above

**3- Home country?..............................................................**

**4- Programme?..........................................................**

BSc. Engg (MCE)

BSc. Engg (CSE)

BSc. Engg (EEE)

BSc. Engg (CCE)

BSc. TVE

BBA

DTVE

MSc. Engg.

PhD

5- How did you get to know about IUT?

1. Social media
2. IUT Website
3. Brochures
4. Relatives
5. Friends
6. Continuing students
7. Formal adverts

**SECTION B:** **ACADEMIC EXPERIENCE**

**Please rate your level of satisfaction with each of the following items about your expectations on Academic Experience at IUT using the following Likert scale;**

(1- Very Dissatisfied. 2- Dissatisfied. 3- Neutral. 4- Satisfied. 5- Very Satisfied)

Lecture rooms 1 2 3 4 5

Quality of teachers 1 2 3 4 5

Course content 1 2 3 4 5

Teaching methods 1 2 3 4 5

Testing methods 1 2 3 4 5

Academic Advising 1 2 3 4 5

Laboratory facilities 1 2 3 4 5

Library services 1 2 3 4 5

University’s automation system 1 2 3 4 5

Timely feedback from teachers 1 2 3 4 5

Is there any other factor (s) you would like to share about your level of satisfaction with **Academic Experience** at IUT?

……………………………………………………………………………………………………………………………………………………………………

**SECTION C:** **SUPPORT SERVICES**

**Please rate your level of satisfaction with each of the following items about your expectations on Support Services at IUT using the following Likert scale;**

(1- Very Dissatisfied. 2- Dissatisfied. 3- Neutral. 4- Satisfied. 5- Very Satisfied)

Car parking space 1 2 3 4 5

Campus accommodation services 1 2 3 4 5

Food services in the cafeteria 1 2 3 4 5

Career counseling & placement 1 2 3 4 5

Medical services 1 2 3 4 5

Communication between students & teachers1 2 3 4 5

University Administrative policies 1 2 3 4 5

Friendliness of IUT staff in time of need 1 2 3 4 5

Wi-Fi free for students 1 2 3 4 5

Support for improving English Language sills1 2 3 4 5

Is there any other factor (s) you would like to share about your level of satisfaction with the **Support Services** at IUT?

……………………………………………………………………………………………………………………………………………………………………

**SECTION D: CAMPUS LIFE**

**Please rate your level of satisfaction with each of the following items about your expectations on Campus Life at IUT using the following Likert scale;**

(1- Very Dissatisfied. 2- Dissatisfied. 3- Neutral. 4- Satisfied. 5- Very Satisfied)

Physical appearance of the campus 1 2 3 4 5

Sports and recreational facilities 1 2 3 4 5

Students’ clubs & organizations 1 2 3 4 5

Students’ own government 1 2 3 4 5

Campus security 1 2 3 4 5

Campus social life 1 2 3 4 5

IUT physical location 1 2 3 4 5

Students’ discipline at campus 1 2 3 4 5

Friendliness of students at campus 1 2 3 4 5

Is there any other factor (s) you would like to share about your level of satisfaction with the **Campus Life** at IUT?

……………………………………………………………………………………………………………………………………………………………………

**SECTION E: ECONOMIC FACTORS**

**Please rate your level of satisfaction with each of the following items about your expectations on Economic Factors at IUT using the following Likert scale;**

(1- Very Dissatisfied. 2- Dissatisfied. 3- Neutral. 4- Satisfied. 5- Very Satisfied)

Financial aid 1 2 3 4 5

Transport services for IUT students 1 2 3 4 5

Tuition cost (fees) 1 2 3 4 5

Students’ pocket allowance 1 2 3 4 5

Distance from home 1 2 3 4 5

Cost of leaving at campus 1 2 3 4 5

Opportunity for casual jobs at campus 1 2 3 4 5

Personal survival at campus 1 2 3 4 5

Is there any other factor (s) you would like to share about your level of satisfaction with **Economic Factors** at IUT?

……………………………………………………………………………………………………………………………………………………………………

**SECTION F: IUT CORPORATE IMAGE/REPUTATION**

**Please rate your level of satisfaction with each of the following items about your expectations on IUT Corporate Image/Reputation using the following Likert scale;**

(1- Very Dissatisfied. 2- Dissatisfied. 3- Neutral. 4- Satisfied. 5- Very Satisfied)

IUT as a subsidiary organ of OIC 1 2 3 4 5

IUT Image in your country 1 2 3 4 5

IUT image in Bangladesh 1 2 3 4 5

Academic reputation of IUT 1 2 3 4 5

Islamic reputation of IUT 1 2 3 4 5

Size of the campus 1 2 3 4 5

Suitable for career preparation 1 2 3 4 5

IUT’s Vision & Mission 1 2 3 4 5

Environment for international exposure 1 2 3 4 5

IUT Reputation for excellent staff 1 2 3 4 5

Is there any other factor (s) you would like to share about your level of satisfaction with **IUT Corporate Image/Reputation**?

……………………………………………………………………………………………………………………………………………………………………

**Thank you very much for your valuable time.**
