# Supplementary material for: Predicting students’ satisfaction with academic services at a multicultural engineering university in Bangladesh: A multiple regression analysis
Source: PLoS One. 2024 Sep 6;19(9):e0309223. doi: 10.1371/journal.pone.0309223 (PMC11379188; doi:10.1371/journal.pone.0309223)
Supplement: Attachment — Submitted filename: Response to Reviewers.docx [file pone.0309223.s003.docx]

**Response to the Reviewers’ comments**

| **Reviewer 1 Comment** | **How it was addressed** | **Location** |
| --- | --- | --- |
| Research objective and question is too much complex. Author should write in simple manner. Author should write research objectives as per the variables. Research questions should rewrite as per objectives.  Each research questions should answer separately in the data analysis and result section.  Author stated that Google form have been used for the data collections so how can author claimed that stratified random sampling have been used in the study ? So clarify the sample selection process. Sample size is appropriate. Author should rewrite research finding section and discussion section. Author should use statistical analysis techniques as per reframe questions. | We extend our gratitude to the esteemed reviewer for their valuable input on our manuscript.  Two hypotheses, denoted as H1 and H2, have been incorporated into section 5, aligning with the research question. This addition aims to provide a more comprehensive understanding of the variables under investigation.  In the analysis section, the results of the hypothesis tests conducted through multiple regression analysis are presented separately. This ensures clarity in demonstrating whether the hypotheses are accepted or rejected.  The study employed a disproportional stratified random sampling approach. This involved dividing the population into distinct subgroups or strata, with a sample selected from each stratum as a single stratum.  A Google Form survey questionnaire was distributed randomly among participants within each stratum, ensuring a representative and diverse sample. | Page 5,6  Yellow  Page 10, 11, 12  Yellow  Page 7  Yellow |
| **Reviewer 2 Comment** | **How it was addressed** |  |
| A. Please restate and structure your abstract in 200 to 300 words based on the manuscript's important ideas. It must include the following sections: (1) introduction to the problem. (2) aims; (3) methods; (4) results; (5) conclusion; and (6) keywords with three parts: (a) discipline of the study; (b) concepts studied; and (c) methodologies and processes.  B. After the conclusions, incorporate this methodology' recommendations into your study. | We thank the reviewer for this comment. We have now restated the abstract in 230 words following the guidelines provide by the reviewer.  Recommendations are now added after the conclusions. | Page 1  Yellow  Page 14  Yellow |
| **Reviewer 3 Comment** | **How it was addressed** | **Location** |
| 1) Please clearly highlight the hypotheses of this research and the methodology used to prove or disprove it.  2) The Abstract section does not highlight the novelty clearly.  3) The introduction section is vaguely written. Introduction section must be written on more qualitative way.  4) English language should be carefully checked and carefully check paper for language typos. There are few typo and grammatical errors in the paper.  5) Organization and formatting of the paper is poor. | We appreciate the reviewer for insightful comments.  1) Two hypotheses, denoted as H1 and H2, have been incorporated into section 5, aligning with the research question. This addition aims to provide a more comprehensive understanding of the variables under investigation.  2) The abstract is now revised and highlighted the novelty.  3) After careful restructuring of the whole article the introduction now reflects the credibility.  4) After a meticulous restructuring of the entire article, we are pleased to inform you that the introduction section reflects its credibility.  5) The organisation and the formatting of the paper is now revised. | Page 5, 6, 9, 11, 12  Yellow  Page 1  Yellow |
