# Supplementary material for: Predicting students’ satisfaction with academic services at a multicultural engineering university in Bangladesh: A multiple regression analysis
Source: PLoS One. 2024 Sep 6;19(9):e0309223. doi: 10.1371/journal.pone.0309223 (PMC11379188; doi:10.1371/journal.pone.0309223)
Supplement: Attachment — Submitted filename: Response to Reviewers.docx [file pone.0309223.s005.docx]

**Response to the Comments of the reviewer**

| **Comments to the Authors** | **How it was addressed** | **Location** |
| --- | --- | --- |
| Primarily, I'd like to extend congratulations to the authors for their choice of article topic and the statistical methodology employed for analysis. I wish to highlight certain areas for improvement.  Given that this is a statistical study involving latent and observable variables, it would have been more appropriate to utilise Structural Equation Modelling (SEM), which offers advantages over regression.  It is essential to present all steps of the SEM for validating the hypotheses. Null hypotheses (Ho) should be provided for each alternative hypothesis (H1 and H2). | We thank the reviewer for their congratulations on our choice of article topic and the statistical methodology employed for our analysis. We appreciate the reviewer's feedback and are grateful for their constructive comments on areas for improvement.  We appreciate the reviewer's suggestion regarding Structural Equation Modelling (SEM) as a potentially advantageous approach for statistical analysis. However, in this particular study, our primary focus was to identify the key factors influencing students' satisfaction in an engineering university through regression analysis.  The collected data and study objectives were specifically tailored towards this goal, aiming to pinpoint the most significant predictor variable among the observed factors. While SEM indeed offers valuable insights by simultaneously considering latent and observable variables, its application might not align with the scope and objectives of our research.  Regression analysis provides a straightforward and widely accepted method for determining the relationship between independent and dependent variables, which suited our study's purpose effectively. Moreover, considering the complexity and resource demands associated with SEM, employing it without clear relevance to our research goals could potentially introduce unnecessary complexity and ambiguity to our findings.  Here are a few examples of research studies conducted in the higher education field that utilized multiple regression analysis (MRA) without employing Structural Equation Modelling (SEM):  Koob, C., Schröpfer, K., Coenen, M., Kus, S., & Schmidt, N. (2021). Factors influencing study engagement during the COVID-19 pandemic: A cross-sectional study among health and social professions students. *PLoS One*, *16*(7), e0255191.  Abdolrezapour, P., Jahanbakhsh Ganjeh, S., & Ghanbari, N. (2023). Self-efficacy and resilience as predictors of students’ academic motivation in online education. *PLoS One*, *18*(5), e0285984.  Tarman, B., & Kilinc, E. (2023). Predicting High School Students’ Global Civic Engagement: A Multiple Regression Analysis. *The Journal of Social Studies Research*, 47(1), 56-63. https://doi.org/10.1016/j.jssr.2022.02.001  Julián, M., & Bonavia, T. (2021). Understanding unethical behaviors at the university level: A multiple regression analysis. *Ethics & Behavior*, *31*(4), 257-269.  While SEM is undoubtedly a powerful tool for certain types of analyses, its application is guided by the specific research questions and objectives at hand. In our case, regression analysis emerged as the most suitable approach for achieving our study's aims of identifying the primary predictor of students' satisfaction in an engineering university. |  |
| It's worth noting that the questionnaire employs an interval scale (ranging from 1 to 5). This detail should be included on text. | The information regarding the interval scale of the questionnaire (ranging from 1 to 5) has been included in the document. Specifically, this detail can be found on page 8, section 6.5, and further elaborated in Appendix A on page 20. | Page 8, section 6.5, Yellow  Page 20, Appendix A  Yellow |
